# Effect of Spermidine on Endothelial Function in Systemic Lupus Erythematosus Mice

**DOI:** 10.3390/ijms25189920

**Published:** 2024-09-14

**Authors:** Hyoseon Kim, Michael P. Massett

**Affiliations:** Department of Kinesiology and Sport Management, Texas Tech University, Lubbock, TX 79409, USA

**Keywords:** MRL/MpJ-Faslpr/J, thoracic aorta, vascular, PINK1–parkin, mitophagy, autoantibodies, vascular cell adhesion molecule 1 (Vcam1)

## Abstract

Endothelial dysfunction is common in Systemic Lupus Erythematosus (SLE), even in the absence of cardiovascular disease. Evidence suggests that impaired mitophagy contributes to SLE. Mitochondrial dysfunction is also associated with impaired endothelial function. Spermidine, a natural polyamine, stimulates mitophagy by the PINK1–parkin pathway and counters age-associated endothelial dysfunction. However, the effect of spermidine on mitophagy and vascular function in SLE has not been explored. To address this gap, 9-week-old female lupus-prone (MRL/lpr) and healthy control (MRL/MpJ) mice were randomly assigned to spermidine treatment (lpr_Spermidine and MpJ_Spermidine) for 8 weeks or as control (lpr_Control and MpJ_Control). lpr_Control mice exhibited impaired endothelial function (e.g., decreased relaxation to acetylcholine), increased markers of inflammation, and lower protein content of parkin, a mitophagy marker, in the thoracic aorta. Spermidine treatment prevented endothelial dysfunction in MRL-lpr mice. Furthermore, aortas from lpr_Spermidine mice had lower levels of inflammatory markers and higher levels of parkin. Lupus phenotypes were not affected by spermidine. Collectively, these results demonstrate the beneficial effects of spermidine treatment on endothelial function, inflammation, and mitophagy in SLE mice. These results support future studies of the beneficial effects of spermidine on endothelial dysfunction and cardiovascular disease risk in SLE.

## 1. Introduction

SLE is a complex, multisystemic autoimmune disorder that is characterized by a loss of tolerance to proinflammatory cytokines and multi-organ damage. A key disease characteristic is the dysregulation of immune complexes associated with low tolerance of proinflammatory responses and uncontrolled autoantibodies [1]. While there is no cure for SLE, the overall mortality rate has improved with treatment with immunosuppressive drugs [2]. However, cardiovascular disease (CVD) remains a leading cause of morbidity and mortality in SLE [3,4]. A 7- to 10-fold higher risk of CVD has been reported in SLE patients, and the prevalence of CV events such as myocardial infarction and coronary artery disease among women with SLE is 50 times higher than age- and sex-matched healthy individuals [5]. Individuals with SLE are susceptible to developing CVD regardless of the traditional CVD factors [5,6]. Because of its complexity and clinical heterogeneity, treatment of SLE is challenging, and current therapies, while effective, are associated with significant complications. To date, there have been no drug targets for both systemic lupus complications and CVD events of SLE. 

Emerging evidence supports that impaired mitochondrial quality control (MQC) contributes to SLE pathogenesis [7,8,9,10]. MQC mechanisms are essential for maintaining the quality and integrity of mitochondria. Mitophagy is one of the components of MQC which has been shown to have a pivotal role in cardiovascular homeostasis [11]. Rodents with mitophagy-related genetic mutations showed compromised vascular homeostasis and endothelial dysfunction, suggesting the importance of mitophagy in vascular health [12,13]. Moreover, impaired endothelial and cardiomyocyte function in aged mice was rescued by enhancing mitophagy [14]. Ineffective clearance of damaged mitochondria (mitophagy) is detrimental to MQC because accumulated damaged mitochondria release a marked amount of Damage-Associated Molecular Patterns (DAMPs), including oxidized mtDNA, cardiolipin, and TFAM, initiating a cascade of inflammatory responses. One of the primary ways to enhance mitophagy is mediated by phosphatase and tensin homolog (PTEN)-induced kinase1 (PINK1) and E3-ubiquitin ligase parkin. The importance of recruiting parkin and parkin-mediated mitophagy was demonstrated in studies that showed accumulated mtDNA mutations and oxidative stress along with a reduced life span in parkin null mice [15,16]. Enhancing the PINK1–parkin pathway has been shown to increase mitophagy [17,18] and improve endothelial function in aging models and diabetic cardiomyopathy [18,19].

Spermidine is a natural polyamine that regulates the PINK1–parkin pathway [20]. Chronic supplementation of spermidine had beneficial effects on eliciting mitophagy, extending the lifespan of mice, and ameliorating renal injuries when applied to salt-induced hypertensive rats [21]. It also had a counteracting effect on age-associated endothelial dysfunction by activating mitophagy and increasing nitric oxide (NO) bioavailability [19]. Other cardioprotective effects of spermidine supplementation include a reduction in chronic inflammation [22,23] and possible improvement in systemic arginine bioavailability [19], which is also favorable for vascular health. However, the role of spermidine on mitophagy and the pathogenesis of endothelial dysfunction in SLE is unknown. Therefore, the purpose of this study was to determine the effect of enhancing PINK1–parkin-mediated removal of damaged mitochondria with spermidine on endothelial dysfunction in SLE. 

Several clinical phenotypes and immune abnormalities seen in patients with SLE are observed in Murphy Roths Large (MRL)/lymphoproliferation (lpr) (MRL/MpJ-Faslpr/J or MRL/lpr) mice. These mice develop immune complex nephritis, skin rashes, aberrant B and T cell responses, increased proinflammatory cytokines, and splenomegaly [24]. Elevated levels of malondialdehyde (MDA) have also been observed in multiple tissues from MRL/lpr mice, suggesting these mice have elevated ROS levels [9]. Most importantly, MRL/lpr mice are considered an excellent model to investigate vascular dysfunction at a relatively early age. Therefore, this mouse strain was used to explore the effects of spermidine in preventing and treating endothelial dysfunction in lupus mice. We observed that spermidine significantly improved endothelial-dependent vasorelaxation and reduced inflammatory responses, suggesting a potential role in reducing CV risk in SLE. 

## 2. Results

### 2.1. Effect of Spermidine on Lupus Phenotypes

To assess if spermidine treatment can prevent the development of SLE phenotypes, both MRL/lpr and MRL/MpJ mice received spermidine via drinking water starting at 9 weeks of age when they had no overt lupus manifestations (Table 1). After 8 weeks of spermidine treatment, no significant difference was found in body weight or heart weight-to-body weight (HW/BW) across the groups (Table 1). There were significant differences in SLE-specific phenotypes. The spleen was significantly longer in the lpr_Control mice compared to the healthy MpJ_Control mice (*p* < 0.0001). Spleen weight was also significantly greater in the lpr_Control mice compared with the MpJ_Control mice when expressed in absolute values (e.g., in mg) (*p* < 0.0001) or relative to body weight (SW/BW, *p* < 0.0001) (Table 1). The result indicates that SLE mice develop splenomegaly, a marker for the lymphoproliferation that occurs in MRL/lpr mice. Nephritis can be assessed by measuring proteinuria and/or by histological diagnosis. We measured urinary protein levels as a general marker of kidney function and disease activity although histological assessment could provide information regarding organ damage and inflammation. The number of mice with urinary protein over 300 mg/dL was six in the lpr_Control mice, while the number was reduced to three in the lpr_Spermidine group. No mouse had over 300 mg/dL of urinary protein in either the MpJ_control or MpJ_Spermidine groups. 

The serum levels of anti-cardiolipin antibodies from lpr_Control were significantly higher than the serum antibody level from MpJ_Control (*p* < 0.0001) (Table 1). The serum anti-dsDNA level was significantly higher in lpr_Control when compared with MpJ_Control (*p* = 0.007), which is consistent with the result of the anti-cardiolipin antibodies. Spermidine treatment attenuated the level of anti-dsDNA antibodies from the serum of lpr_Spermidine when compared with untreated lpr_Control. SLE phenotypes were significantly correlated with the anti-cardiolipin antibodies, with correlation coefficients from 0.62 to 0.87, *p* = 0.0022 to <0.0001 (Table 2). They were also significantly correlated with the anti-dsDNA antibody levels (r = 0.43 to 0.64, *p* = 0.0013 to 0.038). Body weight and heart weight-to-body weight (HW/BW) were not significantly correlated with the anti-cardiolipin and anti-dsDNA antibodies, suggesting that antibody levels reflect SLE disease activity.

### 2.2. Endothelial Dysfunction Was Prevented by Spermidine Treatment in Lupus Mice

**Figure 1:**
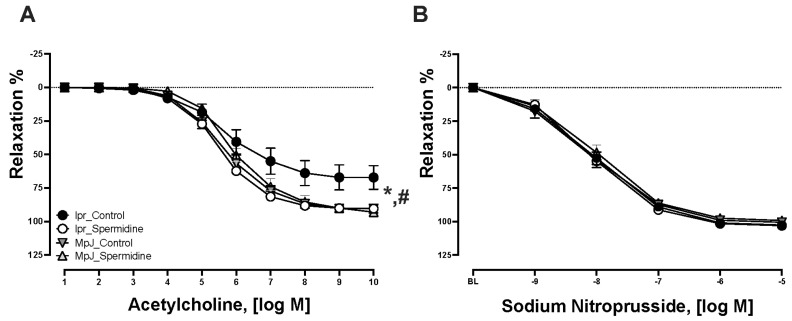
Spermidine prevents endothelial dysfunction in lupus mice. Relaxation responses to increasing concentrations of ACh (**A**) and SNP (**B**) in thoracic aortas from MRL/lpr and MRL/MpJ mice with and without spermidine treatment. Values are mean ± SEM, n = 11–15 per group. BL, baseline after 70% maximal contraction with phenylephrine. * significant difference between lpr_Control and MpJ_Control, *p* < 0.05. # significant difference between lpr_Control and lpr_Spermidine, *p* < 0.05.

Given the beneficial effect of spermidine on vascular function in aging [19], we assessed the functional properties of thoracic aortas from SLE mice treated with spermidine. Thoracic aortas from each group were contracted to 70% of their maximum and were given increasing concentrations of ACh to assess their endothelial function. Concentration–response curves are shown in Figure 1. All vessels relaxed to ACh; however, responses in lpr_Control were significantly reduced (Figure 1A). Maximal responses to ACh were significantly impaired in lpr_Control (67.1 ± 8.7%, n = 11) compared to MpJ_Control (90.3 ± 3.1%, n = 13) (*p* = 0.01), demonstrating that lupus mice had impaired endothelial function. Spermidine improved endothelial function in the thoracic aortas of lpr_Spermidine (90.3 ± 2.8%, n = 13) compared to lpr_Control (*p* = 0.03). Maximal responses to SNP were not significantly different across the groups (Figure 1B). The vasocontractile responses to PE and KCl were measured. There were no significant differences found in the maximal vasocontraction to PE and KCl across any groups.

**Figure 2:**
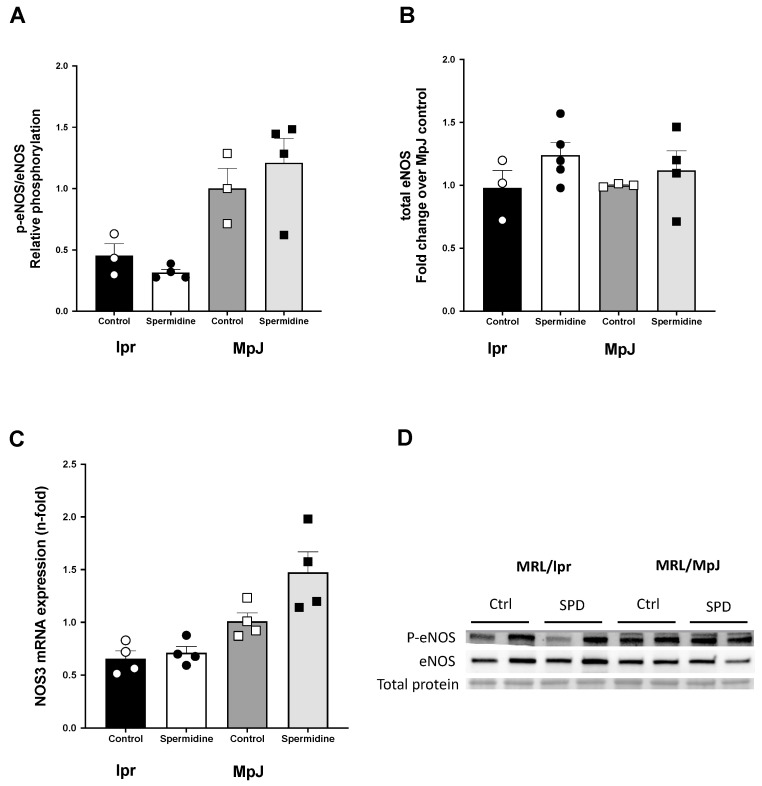
Effect of spermidine on endothelial nitric oxide synthase (eNOS). Densitometry analysis of the Western Blot and mRNA expression for p-eNOS/eNOS (**A**), total eNOS (**B**), and NOS3 (**C**) in thoracic and abdominal aortas from MRL/lpr and MRL/MpJ mice with and without spermidine treatment. (**D**) Western Blot images for phosphorylated eNOS, total eNOS, and total protein by Ponceau stain in thoracic aorta. Values are mean ± SEM. n = 3–5 mice per group.

To determine whether spermidine influences the phosphorylation of eNOS leading to an improvement in endothelial function, we measured eNOS protein in the thoracic aortas (Figure 2). The total eNOS protein level was similar across the groups (Figure 2B,D). Phosphorylation of eNOS at ser 1177 in the aorta from lpr_Control (0.45 ± 0.09 a.u.) relative to the total eNOS was approximately 50% lower than the aortas from MpJ_Control (1.00 ± 0.16 a.u.) (Figure 2A,D), suggesting the potential mechanism of endothelial dysfunction in SLE. The mRNA expression of *Nos3* in the abdominal aortas followed a trend similar to that of phosphorylated eNOS; however, it did not show significant differences across the groups (Figure 2C). Relative gene expression of *Nos3* was 35% lower in lpr_Control (0.65 ± 0.07 a.u.) than in MpJ_Control (1.00 ± 0.07 a.u.), which was consistent with the differences shown in the eNOS phosphorylation in the thoracic aortas.

### 2.3. Anti-dsDNA Antibodies Are Associated with Endothelial Dysfunction

**Figure 3:**
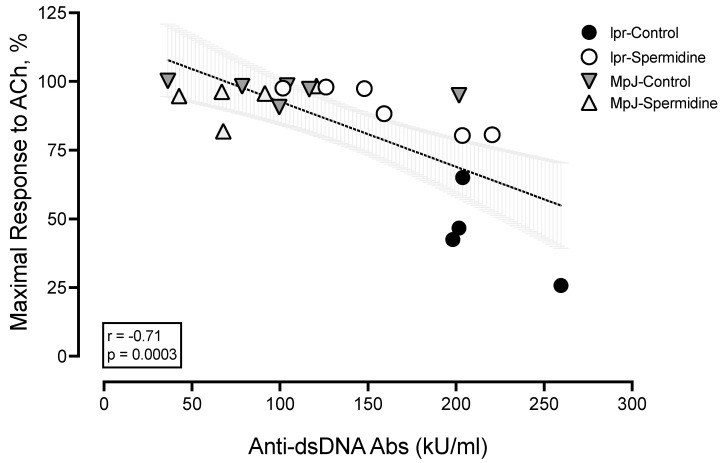
Negative correlation between anti-dsDNA Ab levels and maximal responses to ACh (%). The box contains the correlation coefficient (r) and *p* value.

Maximal vasorelaxation responses to ACh were negatively correlated with anti-dsDNA antibodies (r: −0.71, *p* = 0.0003) (Figure 3). The correlation between maximal vasorelaxation to ACh and anti-dsDNA antibodies was the strongest among all phenotypes (Table 2). This result suggests that the endothelial dysfunction of SLE is associated with disease activities, represented by anti-dsDNA antibodies. Other SLE phenotypes such as spleen weight, length, and kidney weight were all negatively correlated with maximal vasorelaxation response to ACh (r from −0.39 to −0.43 with *p*-value from 0.0049 to 0.0123). Body weight, heart weight, and heart weight-to-body weight were not significantly correlated with ACh-induced vasorelaxation, suggesting that endothelial dysfunction in SLE is related to lupus phenotypes. 

### 2.4. SLE Mice Have Altered Mitophagy in the Aorta and Liver

**Figure 4:**
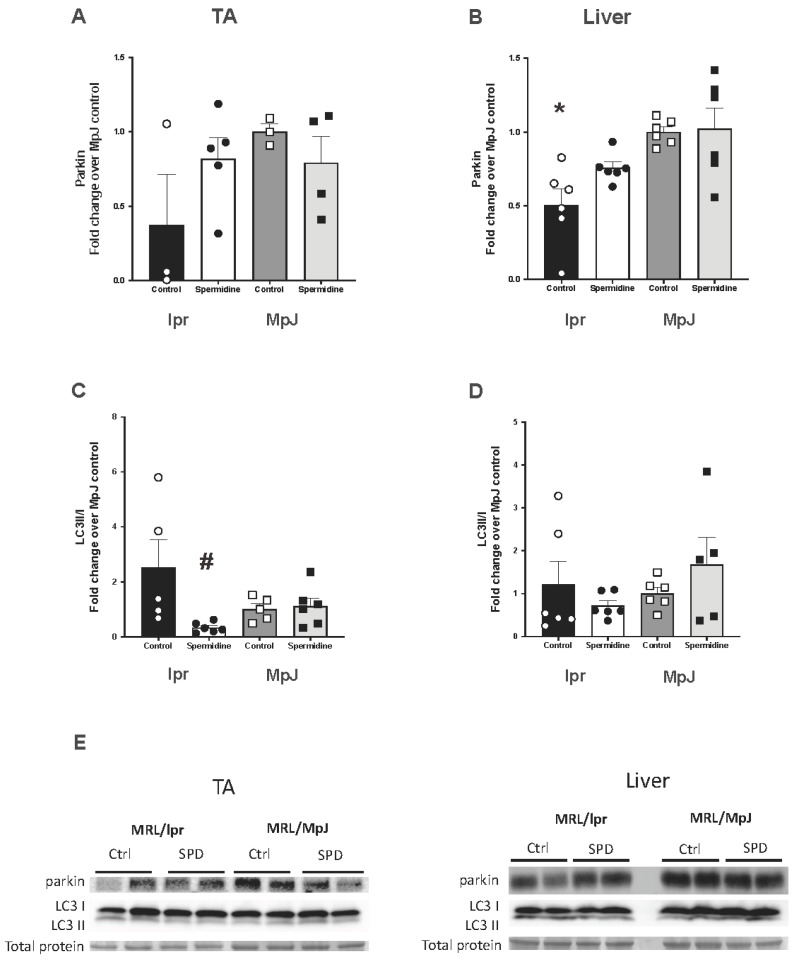
SLE mice have impaired mitophagy in the aorta and liver. Densitometry analysis of the Western Blot for parkin (**A**,**B**) and LC3II/I (**C**,**D**) in thoracic aortas (**left**: **A**,**C**) and livers (**right**: **B**,**D**) from MRL/lpr and MRL/MpJ mice with and without spermidine treatment. (**E**) Western Blot images of parkin, LC3II, LC3I, and total protein by Ponceau stain. Values are mean ± SEM, n = 3–6 mice per group. * significantly different from MpJ_Control, *p* < 0.05. # significantly different from lpr_Control, *p* < 0.05.

Spermidine is considered an activator of mitophagy through the PINK1–parkin pathway. Protein expression of parkin in the thoracic aorta and liver is shown in Figure 4. Parkin protein expression from the thoracic aorta was 62% lower in lpr_Control (0.38 ± 0.34 a.u.) compared to MpJ_Control (1.00 ± 0.05 a.u.), suggesting a potential impaired mitophagy in the aortas of SLE mice (Figure 4A,E). The protein level of parkin was significantly lower in the liver of lpr_Control mice (0.50 ± 0.11 a.u.) compared to healthy control mice, MpJ_Control (1.00 ± 0.03 a.u., *p* = 0.005) (Figure 4B,E). Although the protein levels of parkin in the aorta increased by more than 50% after the spermidine treatment, the treatment did not have a significant impact on the parkin levels in either the aorta or liver of lupus mice. However, the results still suggest that mitophagy is impaired in SLE mice when compared to healthy mice. 

The protein content of LC3 II/I was measured as a marker of mitophagy. The LC3II/I ratio was not statistically different between lpr_Control and MpJ_Control in either the aorta or liver samples. However, both the aorta (Figure 4C,E) and liver (Figure 4D,E) from lpr_Control showed a higher LC3II/I ratio compared to those of MpJ_Control. LC3II/LC3I was significantly lower in lpr_Spermidine (0.33 ± 0.07 a.u.) compared to the lpr_Control group (2.53 ± 0.99 a.u.), which would suggest that spermidine may have increased the mitophagic influx, thus reducing the amount of LC3II/I remaining.

### 2.5. Spermidine Treatment Decreases Inflammatory Markers in Lupus Mice

**Figure 5:**
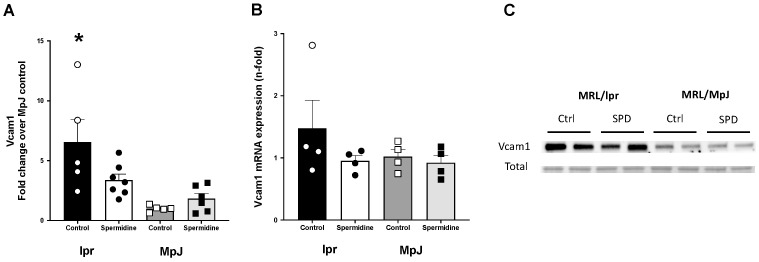
Vascular inflammatory markers are elevated in lupus mice. Densitometry analysis of the Western Blot (**A**) and mRNA expression (**B**) for Vcam1 in thoracic and abdominal aortas from MRL/lpr and MRL/MpJ mice with and without spermidine treatment. (**C**) Western Blot images of Vcam1 and total protein by Ponceau stain. Values are mean ± SEM. n = 4–7 mice per group. * significantly different from MpJ_Control, *p* < 0.05.

Vascular cell adhesion protein 1 (Vcam1) is a marker of inflammation and is associated with atherosclerosis susceptibility [25]. The protein level of Vcam1 from the thoracic aorta was significantly higher in the lupus-prone mice (lpr_Control, 6.55 ± 1.88 a.u., *p* = 0.003) when compared to the healthy control mice (MpJ_Control) (1.00 ± 0.11 a.u.) (Figure 5A,C). The protein content of Vcam1 in the thoracic aorta was 49% higher in lpr_Control compared to lpr_Spermidine (3.38 ± 0.50 a.u., *p* = 0.08), suggesting the potential anti-inflammatory effect of spermidine on SLE. mRNA expression of *Vcam1* in abdominal aorta was higher in lpr_Control (1.48 ± 0.45 a.u.) compared to MpJ_Control (1.00 ± 0.11 a.u.) and lpr_Spermidine (0.95 ± 0.08 a.u.) (Figure 5B).

**Figure 6:**
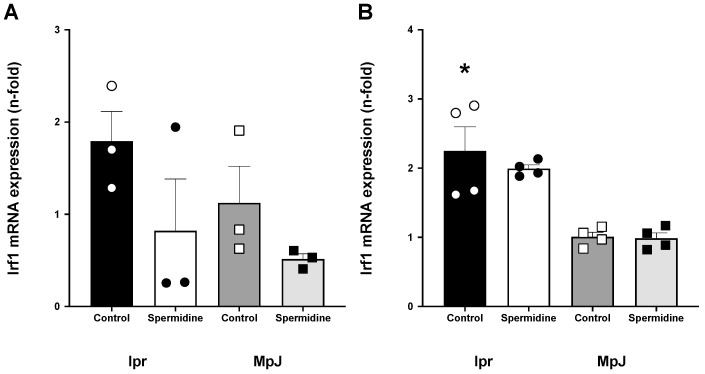
Interferon regulatory factor 1 (*Irf-1*) gene expression in lupus mice. mRNA expression of interferon regulatory factor 1 (*Irf1*) shown in the spleen (**A**) and liver (**B**) from MRL/lpr and MRL/MpJ mice with and without spermidine treatment. Values are mean ± SEM. n = 3–4 mice per group. * significantly different from MpJ_Control, *p* < 0.05.

The protective effect of spermidine related to anti-inflammatory properties is unclear in SLE. We measured the mRNA expression of interferon regulating factor 1 (*Irf1*) in both the spleen and liver (Figure 6). Statistically, there were no differences between the *Irf1* levels in the spleen samples from the lupus-treated and untreated groups, but *Irf1* was 50% lower in the spleen from lpr_Spermidine (0.82 ± 0.56 a.u.) compared to the spleen from lpr_Control (1.79 ± 0.32 a.u.) (Figure 6A). The liver from the lpr_Control group expressed a significantly higher level of *Irf1* mRNA (2.25 ± 0.34 a.u.) compared to the healthy control group, MpJ_Control (1.00 ± 0.06 a.u.) (*p* = 0.002) (Figure 6B). The *Irf1* mRNA level was lower in lpr_Spermidine (1.99 ± 0.05 a.u.) compared to lpr_Control.

## 3. Discussion

Despite the help of immunosuppressant drugs, patients with SLE still struggle with a high risk of CVD. Dietary supplementation with spermidine has been reported to be inversely associated with all-cause mortality and CVD risk in humans [26]. The results of the present study provide novel evidence of the potential effect of spermidine on endothelial dysfunction in SLE. We demonstrated that spermidine, which targets PINK1–parkin-mediated mitophagy, has beneficial effects on lupus mice: (a) improved endothelial function; (b) decreased inflammation; (c) enhanced mitophagy; and (d) did not decrease lupus disease activity. This observation may suggest the significance of this pathway as a therapeutic approach to improve endothelial dysfunction in SLE. 

### 3.1. Spermidine and Endothelial Function

In the current study, we observed a significant strain effect (*p* = 0.007) on maximal endothelium-dependent vasorelaxation. The lupus control group had endothelial dysfunction when compared with the healthy control mice. Two-way ANOVA identified significant effects of intervention with the oral spermidine supplementation (*p* = 0.0075). Eight weeks of treatment with spermidine prevented a decline in endothelial function in lupus mice. Spermidine was previously studied in aged and diabetic mice [19,27]. Both studies demonstrated the effect of spermidine on reversing endothelial dysfunction by improving NO bioavailability. Our data extend previous observations to the effect of spermidine on improving endothelial function in SLE mice. Despite a significant improvement in endothelial function, the molecular mechanisms responsible for the effect are still complex and remain unresolved. In the present study, we measured the phosphorylation level of eNOS protein in the thoracic aorta to investigate the mechanism by which spermidine improved endothelial function in SLE. Phosphorylation of eNOS was 50% lower in lpr_Control when compared to MpJ_Control. However, spermidine did not increase total or phosphorylated eNOS, suggesting that spermidine does not have a direct effect on the NOS activity in SLE. In contrast, Fetterman et al. [27] showed that eNOS phosphorylation and NO production were increased in endothelial cells from diabetic patients by spermidine treatment, suggesting that spermidine can increase eNOS activity. Thus, from the current study and others, the effect of spermidine supplementation on NO production is unclear. Future studies should include multiple measures of NO activity (e.g., serum nitrite levels) to help clarify the mechanisms underlying spermidine’s beneficial effects on endothelial function. 

One of the underlying molecular mechanisms for endothelial dysfunction in the lpr_Control group could be related to the increased level of cell adhesion molecules in the blood vessels of SLE mice. Elevated levels of circulating Vcam1 are associated with endothelial dysfunction [28]. Circulating, urinary, and tissue levels of Vcam1, intercellular cell adhesion molecule 1 (Icam1), and inflammatory cytokines are elevated in MRL/lpr mice and humans with SLE [29,30,31,32]. In the current study, the protein level of Vcam1 was significantly higher in the aortas from lupus mice compared to healthy mice. This is consistent with a previous report of elevated levels of Icam1 from the aorta of lupus mice when compared with healthy control mice [33]. Conversely, the aortas from spermidine-treated mice had significantly lower Vcam1 expression compared to aortas from lupus control mice. Spermidine has been shown in the previous literature to have anti-inflammatory effects, associated with the inhibition of the release of proinflammatory cytokines [34,35]. Our study confirmed the increase in Vcam1 in the aorta of lupus mice, which was reduced by spermidine treatment. To further investigate the potential protective effects of spermidine on vascular function, future studies should consider taking serial measurements of serum Vcam1 with and without spermidine treatment. Changes in circulating Vcam1 are highly correlated with disease activity [29,30] and might provide insight into lupus and CVD progression [36].

The present study measured the mitophagy marker parkin in the vessels of SLE mice. Decreased levels of parkin were shown in both the thoracic aorta and liver from lupus control mice when compared to healthy control mice. Impaired mitophagy is associated with decreased defense mechanisms against inflammation and oxidative stress [37]. The contribution of mitophagy to SLE pathogenesis has been systematically reviewed [38]. However, the exact role of mitophagy in the pathogenesis of SLE requires careful interpretation. Dietary supplementation with spermidine is inversely related to high CVD risks in humans with chronic diseases and is an autophagy/mitophagy-activating compound [21,26,39]. In the current study, the spermidine-treated group had significantly higher parkin levels, suggesting that spermidine induced mitophagy signaling by activating the PINK1–parkin-mediated pathway. Increases in PINK1–parkin signaling and mitophagy are associated with improved endothelial function [18,40], whereas aorta from PINK1 knockout mice have reduced levels of eNOS [40]. Collectively these results support a link between PINK1–parkin signaling, mitophagy/autophagy, and endothelial function. The LC3II/I ratio, one of the markers of mitophagy, was also measured in the blood vessels of lupus mice. We found an increased level of LC3II/LC3I in the lupus aortas, which may suggest the defective clearance of damaged cells. This might suggest that defective mitophagy resulted in the accumulation of LC3 in the cells [41,42]. LC3II/LC3I was significantly lower in the lupus group that was treated with spermidine, suggesting the accelerated turnover of autophagosomes, which carried damaged mitochondria.

The alternative mechanism for improved endothelial function with spermidine treatment in SLE is spermidine acting as an antioxidant. In mouse models of liver disease, spermidine treatment decreased ROS, increased mitochondrial health, and protected endothelial cells from injury [43]. In aging mouse models, spermidine treatment prevented endothelial dysfunction. This improvement in endothelial function was associated with decreased ROS [19]. Excessive ROS play a critical role in the pathogenesis of endothelial dysfunction. An antioxidant, hydroxychloroquine helped improve NO-mediated endothelial function in NZBWF1 lupus mice by potentially reducing NADPH oxidase, the main vascular source of ROS [44]. However, chronic treatment of antioxidants does not change the level of anti-dsDNA antibody levels, the diagnostic marker of SLE [44]. In the present study, it was determined that anti-dsDNA antibody levels have a negative correlation with the maximal endothelium-dependent vasorelaxation of lupus mice and potentially on ACh-induced vasorelaxation. Further study is necessary to determine if spermidine treatment changes the level of ROS, which could potentially affect endothelial function in SLE. 

### 3.2. Spermidine and Inflammatory Responses in Liver and Spleen

In this current study, we observed the level of mRNA expression of interferon regulating factor 1 (*Irf1*) was different between the two strains, suggesting increased stimuli of type 1 interferons (IFNs) and proinflammatory cytokines in SLE mice. This result is comparable with the previous literature, where IFNs were found to be major pathogenic factors in SLE patients and mice [45,46]. Although there have been studies about spermidine eliciting an anti-inflammatory effect on aging and other diseases [23,47,48,49], there have been a limited number of studies assessing the same effect in SLE mice. Our study showed that expression of the *Irf1* gene was lower in both the liver and spleen from the spermidine-treated group compared to the lupus control group, which expands the anti-inflammatory effect of spermidine in SLE. 

### 3.3. Limitations

This study measured both anti-dsDNA antibody and anti-cardiolipin antibody levels. Both are considered as an important parameter for both the diagnosis and treatment of SLE. As expected, both antibody levels were elevated in lupus mice. Eight weeks of spermidine did not change the level of anti-cardiolipin antibodies. However, anti-dsDNA antibody levels were lower after spermidine treatment. This finding is consistent with the negative correlation between anti-dsDNA antibody levels and plasma spermidine levels in SLE patients [50] and supports a potential connection between polyamine catabolism and lupus [50]. Conversely, Kim et al. [51] reported that plasma spermidine levels from SLE patients were not significantly correlated with anti-dsDNA antibody levels or lupus disease activity (e.g., SLEDAI score) [51]. Although the association between plasma spermidine and autoantibody levels is unclear, both studies reported significantly lower plasma spermidine levels in SLE patients than in healthy controls, suggesting a connection between intrinsic spermidine levels and disease activity. The effect of spermidine supplementation in humans on autoantibody levels is unclear. In the current study, spermidine supplementation did not show a significant effect on attenuating other lupus disease phenotypes (e.g., splenomegaly); therefore, spermidine might not have a direct effect on lupus disease activity. 

Although endothelial dysfunction was prevented in spermidine-treated lpr mice, the mechanism for this improvement is unclear. Spermidine treatment had moderate effects on NO signaling, inflammatory, and mitophagy markers. The lack of significant effects on these markers could be related to the dose or route of delivery of spermidine. But spermidine was given via drinking water in a previously reported effective dose [19,21,26,43,52]. This dose and route of delivery was shown to improve endothelial function [19], reduce mitochondrial dysfunction in the mouse aorta [52], reduce mitochondrial oxidative stress and increase autophagy in liver endothelial cells [43], and extend lifespan [21]. Since we did not measure serum or tissue levels of spermidine or its metabolites, it is possible effective levels were not reached in all tissues or in all animals. However, spermidine supplementation in mice [53] and humans [53,54,55,56] did not result in significant increases in tissue [53] or blood levels of spermidine [54,55,56], but was associated with improved inflammatory profiles [54,55] and memory/cognitive function [53,57]. Therefore, the overall beneficial effects of spermidine in humans and our finding of improved endothelial function in mice supports further investigation of spermidine as a potential treatment for endothelial dysfunction in SLE. 

### 3.4. Conclusions

Collectively, spermidine intervention in lupus-prone mice prevented endothelial dysfunction and reduced inflammatory responses in SLE. These findings are novel and provide evidence to support the use of spermidine at early disease onset or as a prophylactic treatment for endothelial dysfunction with SLE. Future studies should explore the effectiveness of spermidine for attenuating or reversing SLE-induced endothelial dysfunction and preventing CVD in SLE patients. 

## 4. Materials and Methods

### 4.1. Ethics Approval

Before starting the study, the study was approved by the Texas Tech University Institutional Animal Care and Use Committee. All procedures were performed under the Public Health Service’s Policy on Human Care and Use of Laboratory Animals guidelines. 

### 4.2. Animal

Female mice from strains MRL/lpr and MRL/MpJ (n = 30/strain) were purchased from Jackson Laboratories (Bar Harbor, ME, USA) and housed in the animal facility at Texas Tech University. Only female mice were used because the disease is somewhat more prevalent and occurs at an earlier age in female mice compared with males [58,59]. All mice were received at 6 weeks of age and were allowed to acclimate for 1–2 weeks prior to use. Five or six mice were housed per cage and were housed in the same room under standard conditions (non-barrier), maintained on a 12:12 h light–dark cycle at a controlled temperature (21–22 °C), and allowed food (Standardize Laboratory Rodent Diet) and water ad libitum. At 8 weeks old, mice were randomly separated into two groups—treatment or control. The treatment group received spermidine added to their drinking water for 6–8 weeks. The control group received normal water. At 16 weeks of age, the mice were weighed and anesthetized by intraperitoneal injection of a cocktail of ketamine (80 mg/kg) and xylazine (5 mg/kg). Serum samples were collected by cardiac puncture. The thoracic aorta, abdominal aorta, heart, kidney, liver, and spleen were harvested for gene expression and Western Blot analysis. Tissues were weighed before being stored. The spleens were removed and their weight and length were measured. The whole tissues were snap-frozen in liquid nitrogen and stored at −80 °C until utilized for the analysis.

### 4.3. Oral (Dietary) Supplementation of Spermidine (SPD) 

Polyamine spermidine (SPD) (3 mM) was administered orally via drinking water. SPD (Sigma-Aldrich, St. Louis, MO, USA, #S0266-5G) was added to the drinking water at a final concentration of 3 mM. Water was replaced every 3–4 days and SPD was freshly added from a 1 M SPD aqueous stock solution (pH 7.4), which was kept at −20 °C for no longer than one month. The 1 M SPD stock solution was prepared by adding 3.14 mL of SPD to a final volume of 20 mL of H_2_O, adjusting the pH to 7.4. Control mice were given regular drinking water. Water consumption was recorded twice a week by weighing the water bottles. 

### 4.4. Urinary Protein

Urine samples were collected from each mouse every week after the initiation of drug treatment to assess proteinuria. Urinary protein excretion was analyzed semi-quantitatively as grade 0 (negative), grade 1+ (<30 mg/dL), grade 2+ (<100 mg/dL), grade 3+ (<300 mg/dL), and grade 4+ (<2000 mg/dL) using a colorimetric assay (Albustix, Siemens Healthcare, Tarrytown, NY, USA) according to the manufacturer’s recommendations. Urine samples with 300 mg/dL or more were defined as proteinuria. 

### 4.5. Vasoreactivity Assessment 

#### 4.5.1. Isolation of Arteries

Thoracic aortas (TAs) were isolated from euthanized mice. Connective tissue and perivascular adipose tissue were carefully removed in an ice-cold physiological saline solution, pH 7.4 (in mM: 118.31 NaCl, 4.69 KCl, 1.2 MgSO_4_, 1.18 KH_2_PO_4_, 24.04 NaHCO_3_, 0.02 EDTA, 2.5 CaCl_2_, and 5.5 glucose), under a microscope. Vessels were cut into 2 mm ring segments of equal length, and each ring segment was suspended in an organ chamber of a 620 M Multi Chamber Myograph System (Danish Myo Technology, Aarhus, Denmark) filled with 8 mL of oxygenated (95% O_2_, 5% CO_2_) physiological saline solution and allowed to equilibrate at 37 °C for at least 30 min. 

#### 4.5.2. Functional Evaluation

Optimal resting tension was determined based on the standard normalization procedures for wire myography [60]. Aortic ring segments were passively stretched in an incremental manner until the calculated transmural pressure reached 13.3 kPa (100 mmHg). Resting tension was set to an internal circumference of approximately 90% of that at 13.3 kPa. Two aortic rings from each mouse were used. The responses from the vessel segments were averaged before statistical analysis. Aorta ring segments were treated with a single concentration of a selective α1 adrenergic receptor agonist, phenylephrine (PE) (10^−6^ M), to confirm that the aorta was viable. A cumulative concentration–response curve to PE (10^−9^–10^−5^ M in full log increments) and potassium chloride (KCl, 5–100 mM) were generated to assess contractile function, whereas a cumulative concentration–response curve to acetylcholine (ACh, a muscarinic receptor agonist) (10^−9^–10^−5^ M in half-log increments) and sodium nitroprusside (SNP, a nitric oxide donor) (10^−9^–10^−5^ M in full-log increments) were generated to assess endothelium-dependent and -independent vasorelaxation, respectively. 

Concentration–response curves to ACh and SNP were generated after each ring was pre-contracted to 70% of their maximum with PE. Percent contraction responses were calculated as [(DP − DB)/DB] × 100, where DP is the maximal force generated by PE and DB is the baseline force. Percent relaxation responses were calculated as [(DP − DD)/(DP − DB)] × 100, where DP is the maximal force pre-generated by PE, DD is the lowest force generated at a given dose of ACh or SNP, and DB is the baseline force. The area under the curve (AUC) for PE and KCl was determined for each vessel as a marker of potency and efficacy using GraphPad Prism 10.

### 4.6. Western Blot

Proteins were isolated from livers and aortic rings (TAs). Tissue lysates were harvested in lysis buffer containing the following: 25 mmol/L Tris-HCl pH 7.5, 150 mmol/L NaCl, 0.1% (*v*/*v*) sodium dodecyl sulphate (SDS), 1% Nonidet-P40 (NP-40), a protease inhibitor cocktail (Roche Applied Science, Barcelona, Spain), and a mix of phosphatase inhibitors (1 mmol/L orthovanadate, 20 mmol/L β-glycerophosphate, 10 mmol/L NaF from Sigma-Aldrich, St. Louis, MO, USA). Protein content was determined with BCA protein assay reagent (Pierce, Rockford, IL, USA), using bovine serum albumin (BSA, Sigma-Aldrich, St. Louis, MO, USA) as standard. Lysates (15–25 μg per lane) were separated by 4–20% mini-protean TGX precast gels and transferred to polyvinylidene difluoride (PVDF) membranes (Bio-Rad Laboratories, Hercules, CA, USA). After protein transfer, the membranes were stained with Ponceau S solution for 15 min to visualize total protein content and verify uniform protein loading. After destaining the membranes, the membranes were blocked with 5% non-fat dry milk in tris buffer solution (TBS) for 1 h at room temperature. Then, the membranes were incubated overnight at 4 °C with monoclonal primary antibodies against parkin (#32833), LC3A/B (#12741), Beclin1 (#3495), Vcam1 (#39036) (1/1000 for each antibody; Cell Signaling, Danvers, MA, USA), p-eNOS (Thr-495, #612706), or eNOS (#610297) (1/1000 for each antibody; BD BioSciences, Becton, NJ, USA). Appropriate HRP-labelled anti-rabbit (1/2500; Santa Cruz Biotechnology, Inc., Santa Cruz, CA, USA) secondary antibodies were subsequently used for 1 h at room temperature. Proteins were visualized using WesternBright Sirius HRP chemiluminescence (Advansta, San Jose, CA, USA) on a Bio-Rad ChemiDoc MP imager (Bio-Rad Laboratories, Hercules, CA, USA). Protein band density was quantified using ImageJ software version 1.54j. Membranes were stripped using ReStore Plus (Thermo Scientific, Waltham, MA, USA) for 1 h 30 min and reblotted with antibodies.

### 4.7. RNA Isolation and cDNA Synthesis

Frozen livers, spleens, and abdominal aortas were homogenized using a FastPrep^®^-24 Instrument with the lysis buffer provided by RNeasy Fibrous Tissue Mini Kit (Qiagen, Valencia, CA, USA). The RNA was then isolated following the manufacturer’s protocol. The RNA concentration was measured with a Nanodrop spectrophotometer (Thermo Scientific, Waltham, MA, USA). RNA (500 ng) was transcribed to cDNA using a High-Capacity cDNA Reverse Transcription Kit (Life Technologies, Grand Island, NY, USA) according to the manufacturer’s protocol. 

### 4.8. Reverse Transcription Quantitative Polymerase Chain Reaction (RT-qPCR)

The real-time PCR was performed as indicated in the user manual with the TaqMan^TM^ Fast Advanced Master Mix for qPCR using the QuantStudio 3 Real-Time PCR systems (ThermoFisher, Waltham, MA, USA). PCR cycle parameters were as follows: 95 °C for 10 min, followed by 40 cycles at 95 °C for 15 s and 60 °C for 1 min. Relative gene expression was calculated using the comparative threshold method (2^−ΔΔCt^). Pre-designed TaqMan^TM^ assays (Thermofisher, Waltham, MA, USA) were used for target genes *Park2* (ID: Mm01323528_m1), *Nos3* (ID: Mm00435217_m1), and *Irf1* (ID: Mm01288580_m1), and housekeeping genes beta-actin (*Actb*) (ID: Mm00486707_m1) (internal normalization) and *Gapdh* (ID: Mm99999915_g1). Data were expressed as fold change normalized to MpJ_Control. 

### 4.9. Enzyme-Linked Immunosorbent Assay (ELISA)

The serum samples were collected by cardiac puncture. Mice serum samples were diluted 1:100 with assay buffer for anti-dsDNA antibody and anti-cardiolipin antibody detection. A Mouse Anti-dsDNA IgG ELISA kit and Mouse Anti-cardiolipin IgG ELISA Kit (#5120 and #5515, respectively, Alpha Diagnostic Intl. Inc., San Antonio, TX, USA) were used following the manufacturer’s instructions. 

### 4.10. Statistical Analysis

An a priori power analysis was conducted using G*Power to detect significant differences (*p* < 0.05, 1 − β = 80%) in responses to ACh (1) between lpr and MpJ strains; and (2) between the lpr treatment and control groups. Based on preliminary data and the literature, n = 4 mice/strain are needed to detect a 30% difference between MRL/lpr and MRL/MpJ, and n = 13 mice/group are needed to detect a 15% difference between the treatment and control groups. Concentration–response data were compared using two-way repeated-measures ANOVA followed by Šídák’s multiple comparisons test. Differences in body weight, heart, and spleen weight, as well as relative gene and protein expression across control groups of each strain or groups within a strain, were analyzed using two-way ANOVA (strain × group), followed by Tukey’s post hoc test to examine the interaction between strain and treatment. Outliers were detected using the ROUT method (Q = 1%) and removed if identified. All statistics were performed using Prism 10 (GraphPad Software, San Diego, CA, USA) or JMP Pro 16 (SAS, Cary, NC, USA). Statistical significance was set at *p* < 0.05.

## Figures and Tables

**Table 1:** Body mass, tissue mass, and autoantibodies in untreated and treated lupus-prone and healthy control mice.

	lpr_Con	lpr_SPD	MpJ_Con	MpJ_SPD
Body weight (g)	40.4 ± 1.1	41.7 ± 1.5	43.0 ± 0.9	40.9 ± 1.5
HW/BW (mg/g)	4.76 ± 0.28	4.49 ± 0.13	4.12 ± 0.08	4.11 ± 0.13
Spleen weight (mg)	613 ± 73	486 ± 52	97 ± 3 *	95 ± 3 *
SW/BW (mg/g)	15.49 ± 1.96	11.92 ± 1.23	2.33 ± 0.10 *	2.35 ± 0.09 *
Spleen length (cm)	2.8 ± 0.2	2.8 ± 0.1	1.8 ± 0.1 *	1.8 ± 0.1 *
Kidney weight (mg)	292 ± 17	269 ± 14	216 ± 5 *	206 ± 5 *
Proteinuria, n/group	6/13	3/12	0/14	0/14
Anti-cardiolipin Abs (kU/mL)	1.20 ± 0.22	1.36 ± 0.30	0.27 ± 0.05 *	0.18 ± 0.05 *
Anti-dsDNA Abs (kU/mL)	199.7 ± 17.5	176.5 ± 23.3	106.1 ± 22.3 *	74.8 ± 11.2 *

BW, body weight; HW/BW, heart weight-to-body weight ratio; SW/BW, spleen weight-to-body weight ratio; Proteinuria, number of mice per group with urine protein samples ≥ 300 mg/dL; Abs, antibody levels measured in mouse serum by antibody-specific ELISA; Con, control mice not receiving spermidine; SPD, spermidine-treated mice; lpr, MRL/lpr lupus-prone mice; MpJ, MRL/MpJ healthy control mice; n = 12–14/group for anthropometric variables; n = 6/group for autoantibodies; data are mean ± SEM; * significantly different from lpr_Control (*p* < 0.05). Spermidine treatment did not significantly reduce lupus phenotypes in lpr mice.

**Table 2:** Correlation (r) between SLE parameters and anti-cardiolipin and anti-dsDNA antibodies.

Variables	Anti-Cardiolipin Abs r, (*p*-Value)	Anti-dsDNA Abs r, (*p*-Value)
Spleen weight-to-body weight	0.79, (<0.0001)	0.64, (0.0013)
Spleen length	0.83, (<0.0001)	0.62, (0.0035)
Kidney weight	0.62, (0.0022)	0.43, (0.0378)
ACh Max	−0.21, (0.3861)	−0.71, (0.0003)

Correlations were performed using data from all animals (n = 6/group). Serum auto-antibodies were positively correlated with lupus disease-related phenotypes and negatively correlated with endothelium-dependent vasorelaxation.

## Data Availability

The data presented in this study are available upon request from the corresponding author.

## Abbreviations

Abs	Antibodies
ACh	Acetylcholine
BW	Body Weight
CVD	Cardiovascular Disease
DAMPs	Damage-Associated Molecular Patterns
eNOS	Endothelial Nitric Oxide Synthase
HW	Heart Weight
Icam1	Intercellular Cell Adhesion Molecule 1
IFNs	Type 1 Interferons
Irf1	Interferon Regulating Factor 1
KCl	Potassium Chloride
LC3	Microtubule-Associated Proteins 1A/1B Light Chain 3A
lpr	MRL/MpJ-Faslpr/J or MRL/lpr mice
MDA	Malondialedehyde
MpJ	MRL/MpJ mice
MQC	Mitochondrial Quality Control
mtDNA	Mitochondrial DNA
NADPH	Nicotinamide Adenine Dinucleotide Phosphate
NO	Nitric Oxide
PE	Phenylephrine
PINK1	Phosphatase and Tensin Homolog (PTEN)-Induced Kinase 1
ROS	Reactive Oxygen Species
SLE	Systemic Lupus Erythematosus
SNP	Sodium Nitroprusside
SPD	Spermidine
SW	Spleen Weight
TA	Thoracic Aorta
TFAM	Mitochondrial Transcription Factor A
Vcam1	Vascular Cell Adhesion Cell 1
